# Supplementary material for: In situ 3D bioprinting with bioconcrete bioink
Source: Nat Commun. 2022 Jun 23;13:3597. doi: 10.1038/s41467-022-30997-y (PMC9225998; doi:10.1038/s41467-022-30997-y)
Supplement: Supplementary file 1 — Supplementary Information [file 41467_2022_30997_MOESM1_ESM.pdf]

Supplementary Information

In Situ 3D Bioprinting with Bioconcrete Bioink

Supplementary Note 1 | Superiority of in-situ bioprinting compared to in-vitro bioprinting

Compared to organ implantation based on traditional 3D bioprinting in vitro, in-situ bioprinting has more advantages for its in-situ deposition feature (Supplementary Fig. 1).

i) In-situ bioprinting can utilize a more complicated and all-round stimulation environment in vivo to facilitate deeper functionalization of the bioprinted structures, whereas in terms of bioprinting in vitro, the bioprinted structures have to be cultured and induced in vitro, so that the functionalization level can be low with current inducing technology in vitro.

ii) In vitro bioprinted structures can be out of shape after culturing because of swelling and degradation. When the structures are implanted, it can be difficult to match the shape of the organ defect, rendering it unimplantable. By comparison, the shape suitability can be high in in-situ bioprinting.

iii) During the transfer process, the bioprinted structures can be broken after relevant post-processes (i.e., grabbing samples with tweezers), which are avoided in in-situ bioprinting.

iv) In in-situ bioprinting, the bioink is crosslinked directly to the organ defect. The bioink infiltrates the wound surface and spontaneously fills in the tiny gaps. After crosslinking, the internal friction between the bioprinted structures and the wound surface is high. In terms of hydrogel-based bioink, hydrogen bonds between the interface are strong, further enhancing their combination, which has been confirmed by previously published studies. However, in vitro bioprinted structures are crosslinked in vitro, so that they have a determinate outline before implanting, and the binding force between the printed structure and the wound is low.

v) The flexibility of in situ bioprinting is high. Even without a professional 3D bioprinter, manual operation or direct injection of the bioink can be an alternative strategy in some urgent situations (battleground, traffic accident), which is daunting in in-vitro bioprinting.

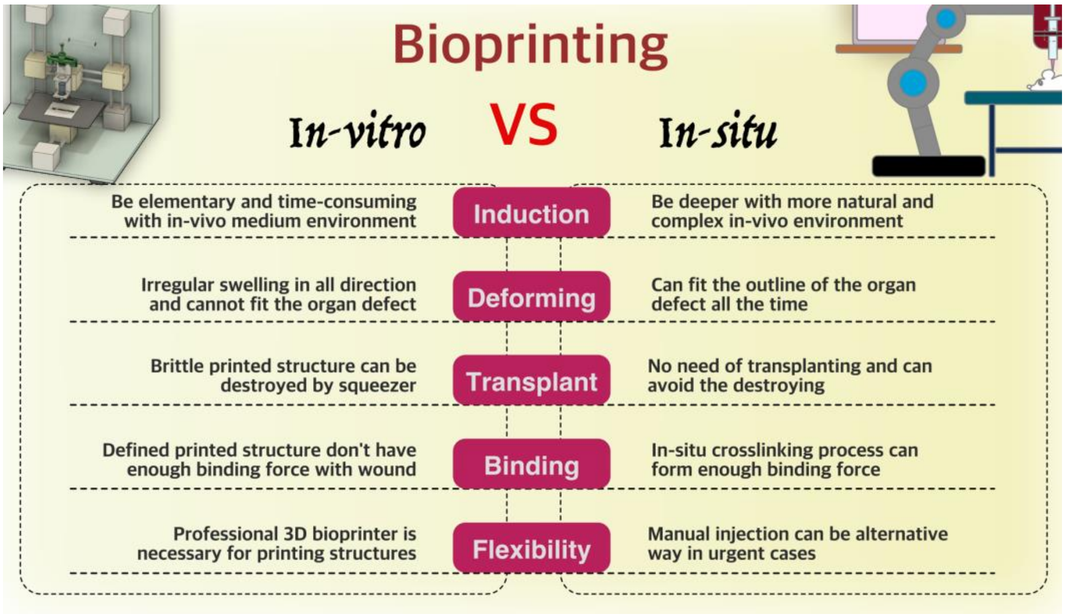

Supplementary Fig. 1 | Superiority of in-situ bioprinting compared to in-vitro bioprinting

Supplementary Note 2 | Calculation method of volume proportion

Due to the scanning layers are discrete and limited with confocal fluorescent microscope, assuming that the volume of A/C in the structure was the sum of elementary volumes of each layer (Supplementary Fig. 2)

The elementary volume of A/C component $\Delta V_{A(C)i}$ was calculated as below:

$$\Delta V_{A(C)i} = S_{A(C)i}\Delta h \tag{1}$$

$S_{A(C)i}$ were analyzed with ImageJ with the confocal fluorescent microscope images. The total volume of A/C component $V_{A(C)}$ was calculated as below:

$$V_{A(C)} = \sum_{i=1}^{layer\_number} \Delta V_{A(C)i} = \sum_{i=1}^{layer\_number} \Delta S_{A(C)i}\Delta h \tag{2}$$

The volume proportion of A component in A-C bioink P_A was calculated as below:

$$P_A = \frac{V_A}{V_A + V_C} = \frac{\sum_{i=1}^{layer_{number}} \Delta S_{Ai}\Delta h}{\sum_{i=1}^{layer_{number}} \Delta S_{Ai}\Delta h + \sum_{i=1}^{layer_{number}} \Delta S_{Ci}\Delta h} = \frac{\sum_{i=1}^{layer_{number}} \Delta S_{Ai}}{\sum_{i=1}^{layer_{number}} \Delta S_{Ai} + \sum_{i=1}^{layer_{number}} \Delta S_{Ci}} (100\%) \tag{3}$$

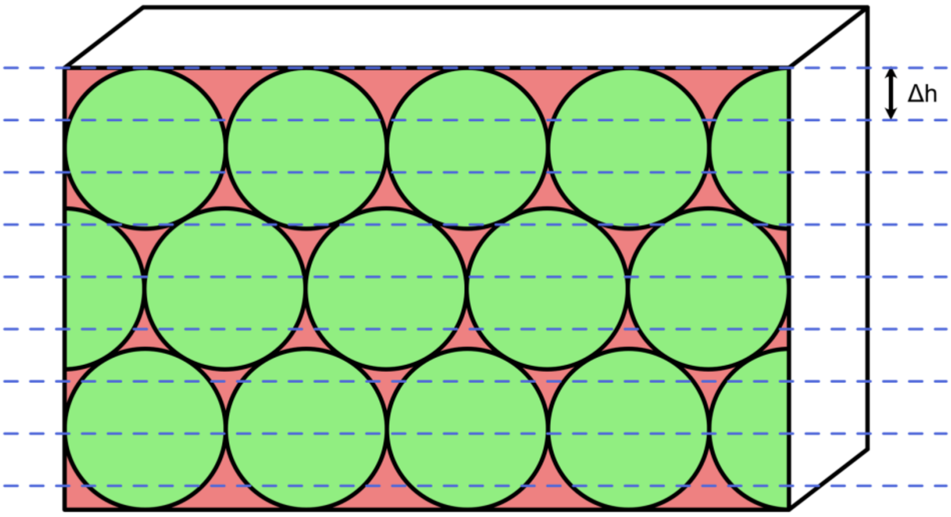

Supplementary Fig. 2 | Calculation model of A/C volume proportion

Supplementary Note 3 | COMSOL simulation of the electrosprayed microdroplet diameter

In our previous research, we have summarized four electrospraying states in GelMA microdroplet fabrication, namely microdripping state, Taylor jetting state, oscillating jetting, multi dripping state. Among them, microdripping state could generate the microgels with uniform diameter. To ensure the excellent printability of A-C bioink, this state was chosen to generate A component. Here, with COMSOL Multiphysics, the relationship between critical diameter of GelMA microdroplet and the applied voltage was analyzed:

$$d_{droplet} = f(U) \quad (4)$$

In terms of the microdroplet at the tip of the nozzle, the mechanical balance model was built (Supplementary Fig. 3).

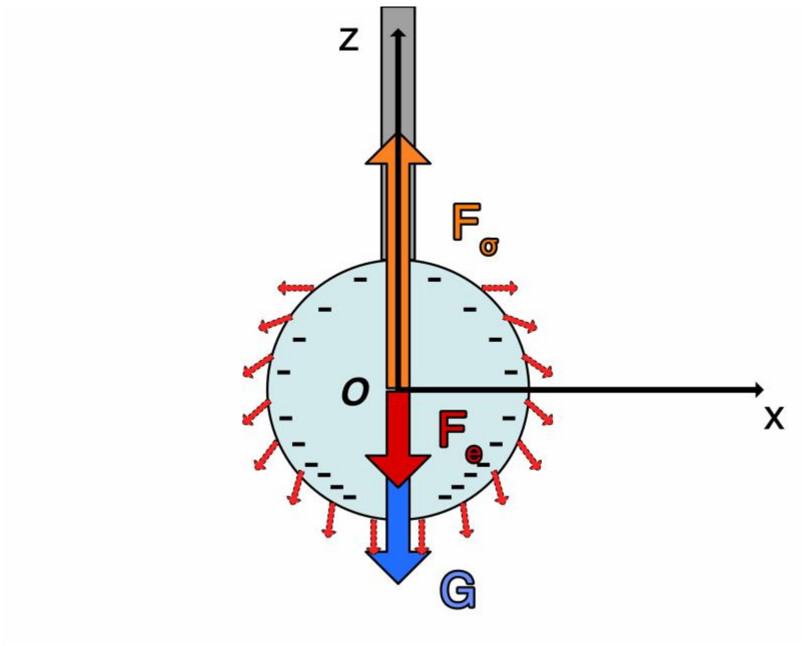

Supplementary Fig. 3 | Mechanical balance model in electrospraying process

The microdroplet was simplified as a standard globe, which was affected by gravity G, the resultant force of electric field force in Z direction F_e, and the surface tension between droplet and nozzle tip F_σ. The mechanical balance equation was as below:

$$G + F_e = F_\sigma \quad (5)$$

$$\oiint \mathbf{E} \cdot \rho_{charge}(x, y, z) \cdot d\mathbf{s} = \pi D_{nozzle-O}\gamma - \frac{\pi}{6} {d_{droplet}}^3 \rho_{e-ink} g \quad (6)$$

where ρ_{e-ink} was the density of electrospraying ink (30 °C, measured as 1100.645 kg/m^3), g was acceleration of gravity (9.8 m/s^2), $\mathbf{E}$ was the surface field strength of GelMA microdroplet, $\rho_{charge}(x, y, z)$ was the point electric density on the microdroplet surface, $D_{nozzle-O}$ was the outer diameter of the electrospraying nozzle, γ was the surface tension coefficient (30 °C, measured as 55.91 mN/m).

Firstly, to explore the uniformity of the surface electric density on the microdroplet at the nozzle tip, regardless of the relationship between critical diameter and applied voltage, the simulation results were acquired with COMSOL Multiphysics (Supplementary Fig. 4). The electric potential on the metal ring was set as 3 kV. The nozzle was chosen as 30G. The GelMA microdroplet was regarded as conductor together with the metal nozzle and connected to the ground because of an ocean of free ions existing in the GelMA precursor solution.

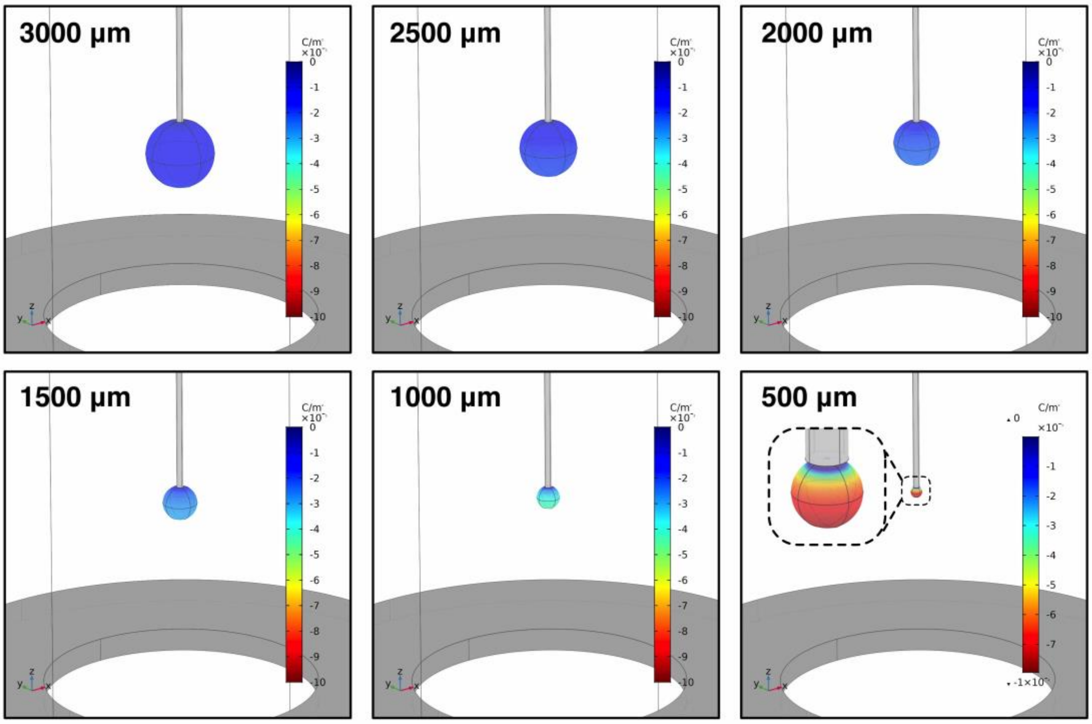

Supplementary Fig. 4 | Simulation of surface electric density distribution on the GelMA microdroplet

From the simulation results, the surface electric density didn't distribute in a uniform way on the microdroplet surface and it showed more obvious with the decrease of microdroplet diameter. It was because the microdroplet attached to the nozzle tip and became a part of the electrode. With the accumulation of microdroplet, the distribution of electric field varied. Thus, there was complex coupling relationship among the applied voltage, critical diameter and the surface electric density, so that it was difficult to get the analytical expression of the resultant force of electric force in Z direction.

Therefore, electric field composed of 27G, 28G, 30G metal nozzle were established respectively and added different voltages (0.5 kV, 1.0 kV, 1.5 kV, 2.0 kV, 2.5 kV, 3.0 kV, 3.5 kV). The microdroplet diameters were set as 500 μm, 1000 μm, 1500 μm, 2000 μm, 2500 μm, 3000 μm. The static electric filed simulation was carried out with COMSOL and the Maxwell electrical surface stress tensors was calculated with surface integral to directly get the resultant force of electric force in Z direction under different applied voltages and microdroplet diameters (Supplementary Fig. 5).

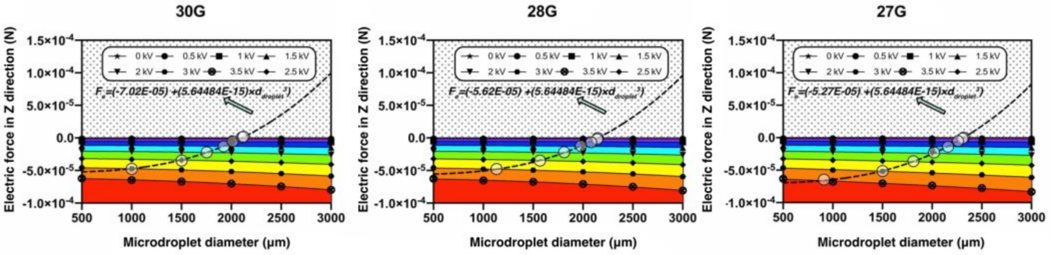

Supplementary Fig. 5 | Simulation results of electric field force in Z direction of the microdroplets with different diameters under different applied voltages

Fitting the $F_e - d_{droplet}$ curve with curve of the second degree and results were shown in Supplementary Table. 1, which demonstrated that the goodness of fit was high.

Supplementary Table. 1 | Fitting goodness of $F_e - d_{droplet}$ curve with curve of the second degree

Nozzle	Voltage U						
size	0.5 kV	1.0 kV	1.5 kV	2.0 kV	2.5 kV	3.0 kV	3.5 kV
**30G**	0.9983	0.9984	0.9984	0.9988	0.9978	0.9984	0.9988
**28G**	0.9973	0.9975	0.9978	0.9980	0.9980	0.9971	0.9976
**27G**	0.9871	0.9905	0.9900	0.9904	0.9900	0.9911	0.9902

The nozzle sizes of different nozzle types were respectively introduced into Equation.(6) and the curves were drawn in Supplementary Fig. 5, which would intersect with the $F_e - d_{droplet}$ curve at certain point. The physics meaning of these intersection points is that the mechanical balance equation and the simulation results could be met at the same time, namely the numerical solution of $f(U)$. By comparison, in three different static electric field models, the diameter of microdroplet decreased with the increase of applied voltage. In the models established with 27G nozzle, the $F_e - d_{droplet}$ curve under 3.5 kV still own intersection point with the mechanical balance equation curve. However, the one established with 28G and 30G nozzle, this intersection points disappeared. It demonstrated that the larger nozzle owns bigger critical voltage.

Supplementary Note 4 | Diameter distribution of microgels diameter

The size of the microgel determines its material exchanging degree with outer environment, which is extremely important for the growth of the encapsulated cells. To explore the microdroplet diameter affected by the electrospraying parameters, orthogonal experiments were carried out with different applied voltages, flowrates and nozzle size.

The simulation results and the data of orthogonal experiments (50 microgels were measured in each group) were summarized and fitted with curve of the third degree. The fitting curves were shown (Supplementary Fig. 6a, Supplementary Table. 2). The diameter decreasing regulation were corresponding to the ones of simulation results. The diameter range of the generated microgels was 492–2310 μm. Under the same electrospraying parameters, the diameters of the microgels were extremely uniform. With the increase of applied voltage, the microgel diameters gradually decreased and the changing rate increased. Besides, the influence of the nozzle on the microgel diameters was obvious. With the same flowrate and applied voltage, bigger microgel diameter would be generated with bigger nozzle. However, the influence of the flowrate on the microgel diameters was not obvious. It was worthy to be mentioned that the curves acquired from experiments were below the ones from simulations. It was because the microdroplet was regarded as standard globe in simulation while it was gradually stretched in reality, so that the connecting effect between the microdroplet and the nozzle tip would be weakened. Furthermore, the stress of electric force in X/Y direction on the microdroplet surface would make the droplet tend to break out, which

further weakened the connection. Therefore, under the same applied voltage, the critical voltages of the orthogonal experiments would decrease compared to the ones in simulation. Microgels with diameter of 500 μm were chosen as A30/5 (Supplementary Fig. 6b).

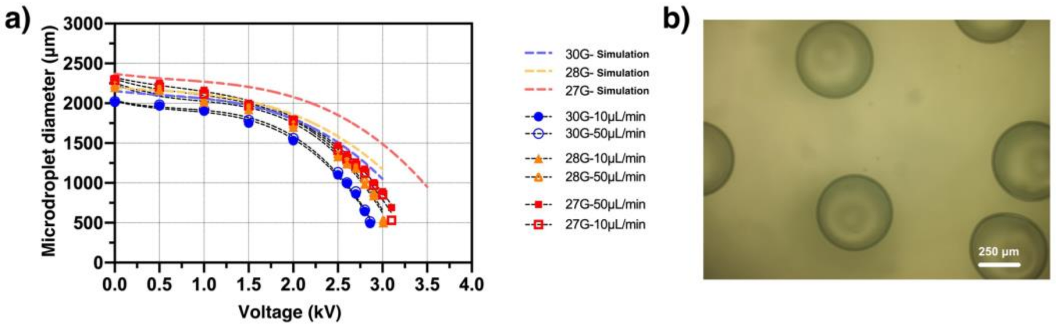

Supplementary Fig. 6 | Fitting of the simulation and experiments of microdroplet diameter and image of chosen A30/5 (500 μm). a) Fitting of the simulation and experiments of microdroplet diameter. b) Image of chosen A30/5 (500 μm). For b), each experiment was repeated independently with similar results for 3 times.

Supplementary Table. 2 | Fitting goodness of $U - d_{droplet}$ curve with curve of the third degree

Nozzle size	Simulation	Experiment	
		10 μL/min	50 μL/min
**30G**	0.9987	0.9963	0.9965
**28G**	0.9994	0.9901	0.9901
**27G**	0.9975	0.9950	0.9983

Supplementary Note 5 | Breakage of microgels during extruding and printing window

The integrality of the microgels in A-C bioink should be strictly guaranteed in case the liquid GelMA precursor solution would enter the microgels from the fracture and fail to build composite structures with clear boundary. Because of the shear stress while the A-C bioink was extruded from the bioprinting nozzle, brittle GelMA microgels could be easily broken. Thus, suitable flowrate and nozzle size range were significant for the microgels with different diameters. The testing results were shown (Supplementary Fig. 7).

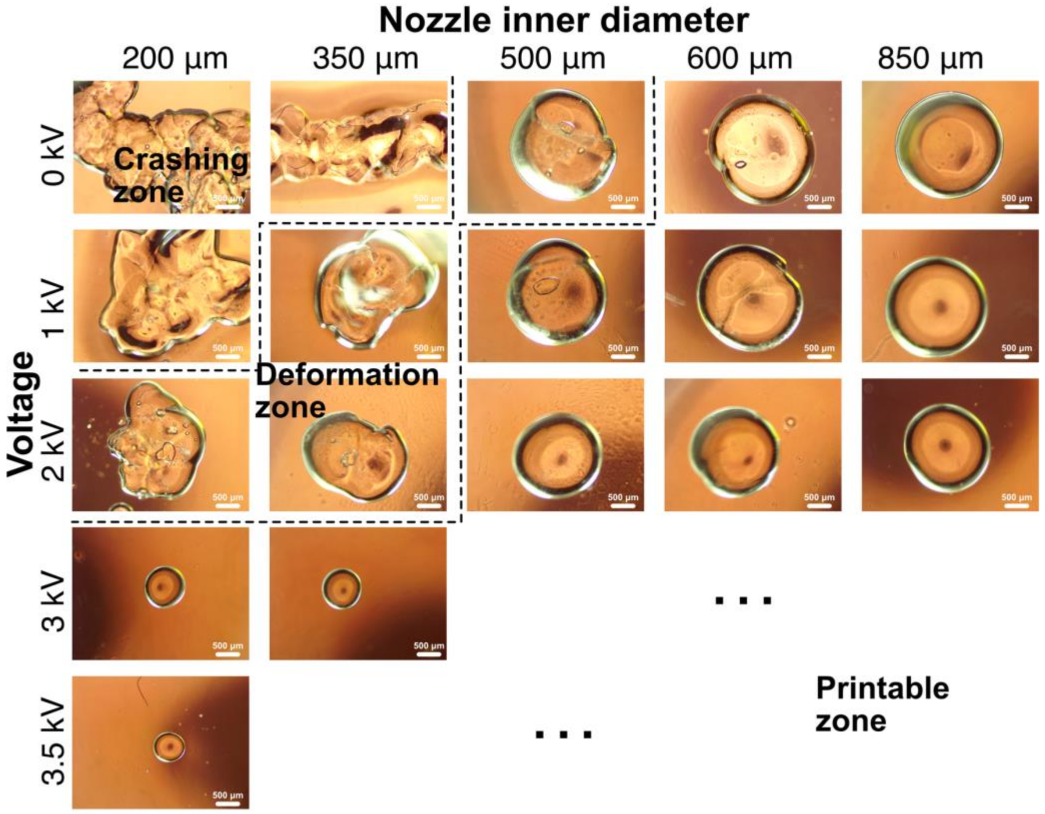

Supplementary Fig. 7 | The integrality of the microgels in A-C bioink

From the result, we could find that the difference between microgel diameter and nozzle inner diameter would obviously influence the integrality of the A component. In actual in-situ bioprinting, the choice of nozzle type should be strictly selected according to the diameter of applied microgels.

Supplementary Note 6 | Scanning electron microscope (SEM) morphology of C/A-C structures

The micro-scale morphology of C/A-C structure was captured with SEM (Supplementary Fig. 8). Compared to the pure C structure, clear boundary between microgels and precursor solution could be found in A-C structures, which demonstrated the formation of the composite structure. Larger micropores could be found in the area of microgels (A30/5) and denser micropores could be found in the area of C component.

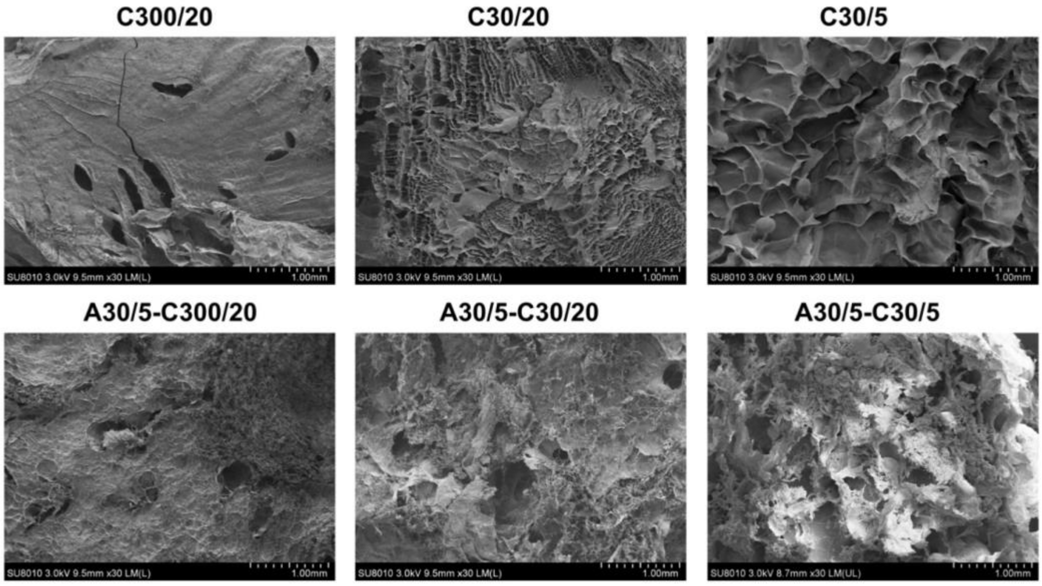

Supplementary Fig. 8 | SEM morphology of C/A-C structures

Supplementary Note 7 | A component encapsulating other cell species

Apart from the stem cells like BMSCs applied in this paper, much more application situations could need different cell species for different therapeutic effect. Here, MC3T3-E1s-laden (somatic cells in early differentiation state), HUVECs-laden (somatic cells in late differentiation state) and MDA-MB-231s-laden (tumor cells) A components were respectively electrosprayed (Supplementary Fig. 9–11). The viabilities of the cells showed to be high and the actin morphology showed that the cells could spread in the 3D microenvironment, which verified that different cell species could be further applied in the establishment of A-C bioink in different therapy situations.

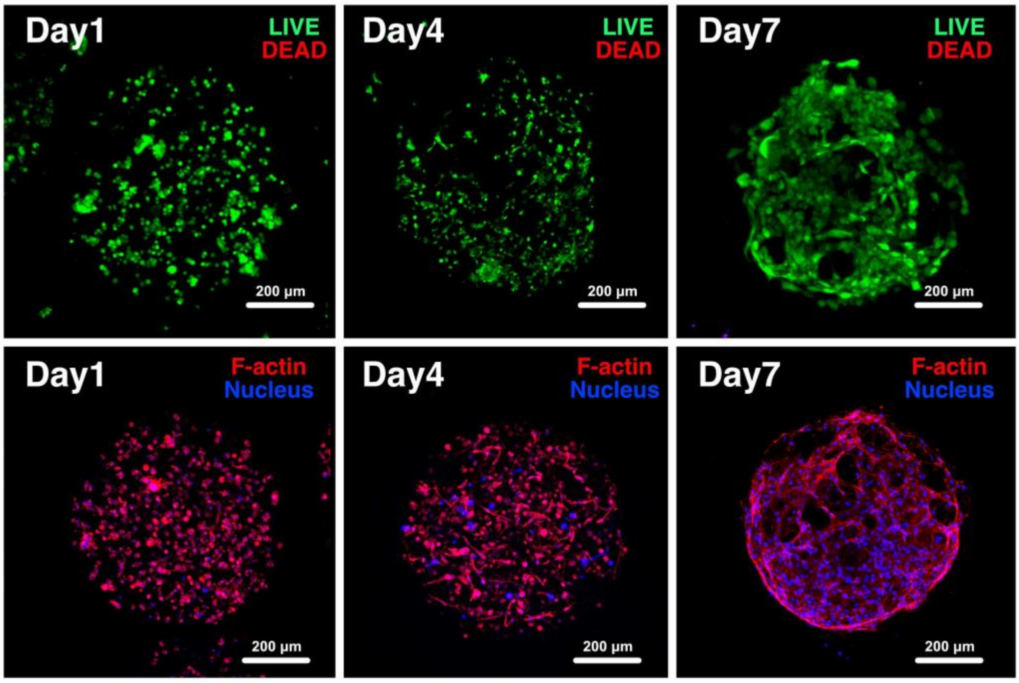

Supplementary Fig. 9 | Viability and actin morphology of MC3T3-E1s-laden A component. Each experiment was repeated independently with similar results for 3 times.

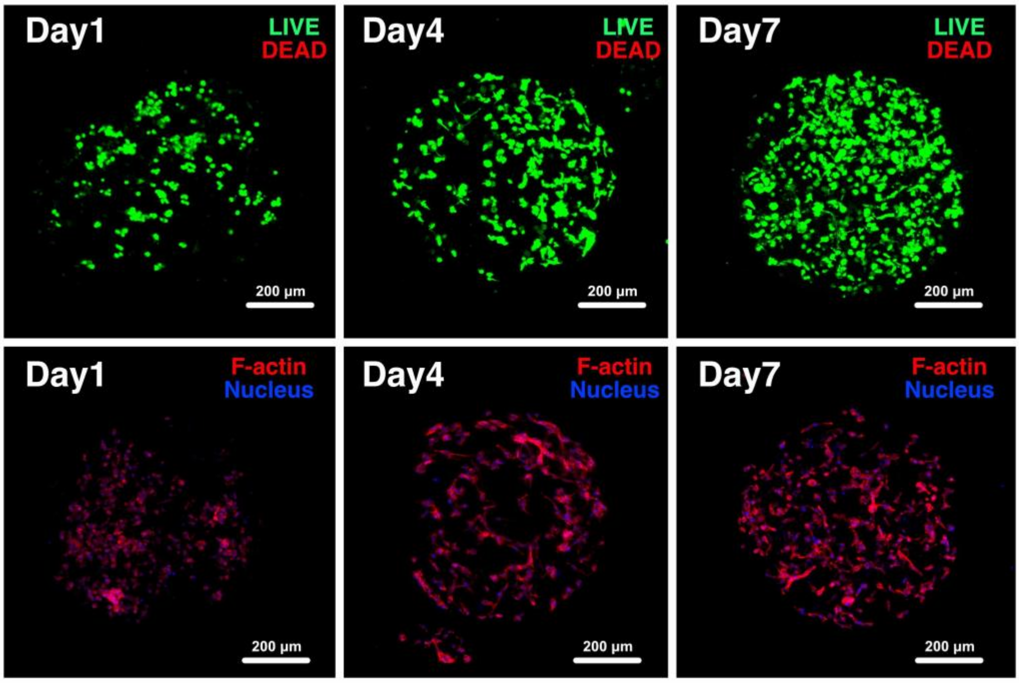

Supplementary Fig. 10 | Viability and actin morphology of HUVECs-laden A component. Each experiment was repeated independently with similar results for 3 times.

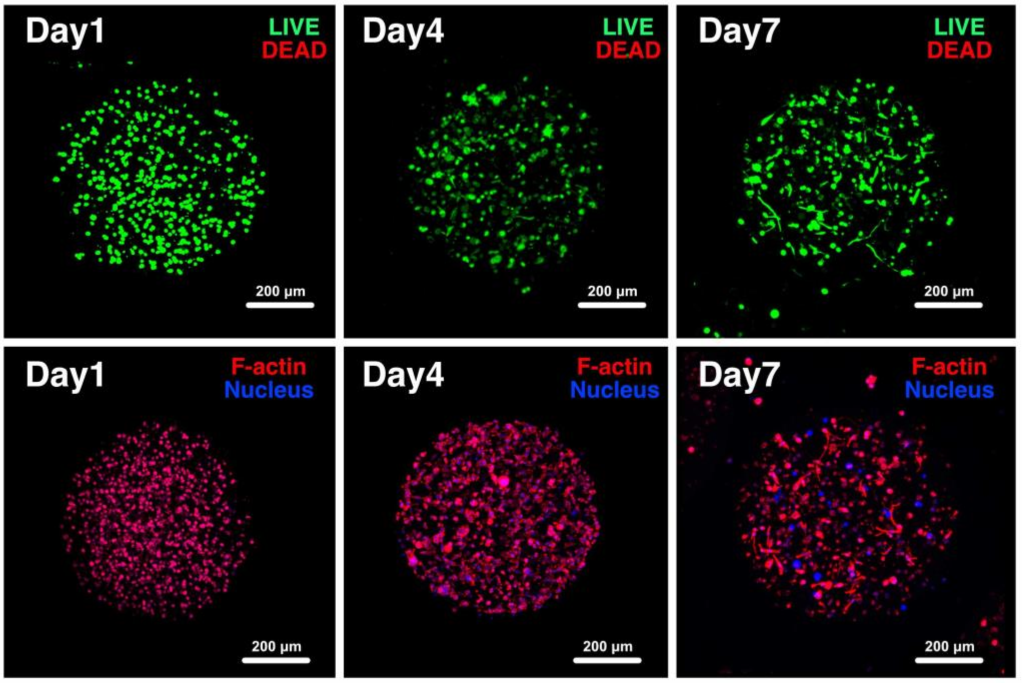

Supplementary Fig. 11 | Viability and actin morphology of MDA-MB-231s-laden A component. Each experiment was repeated independently with similar results for 3 times.

Supplementary Note 8 | Outlook of cell co-culturing with complicated A-C bioink

As discussed in the DISCUSSION, more complex therapy situations could happen, in which the vascularization in in-situ bioprinting could be required. To examine the vascularization capability in A-C bioink system, HUVECs-laden A components were electrosprayed and mixed with MDA-MB-231s-laden C component. Because of the VEGF releasing ability of MDA-MB-231s, HUVECs would tend to rapid grow and gradually form 3D sprout. 3D sprout from A component formed after 5-day co-culturing, which demonstrate the potential of vascularization capability of A-C bioink for further complicated therapy cases (Supplementary Fig. 12).

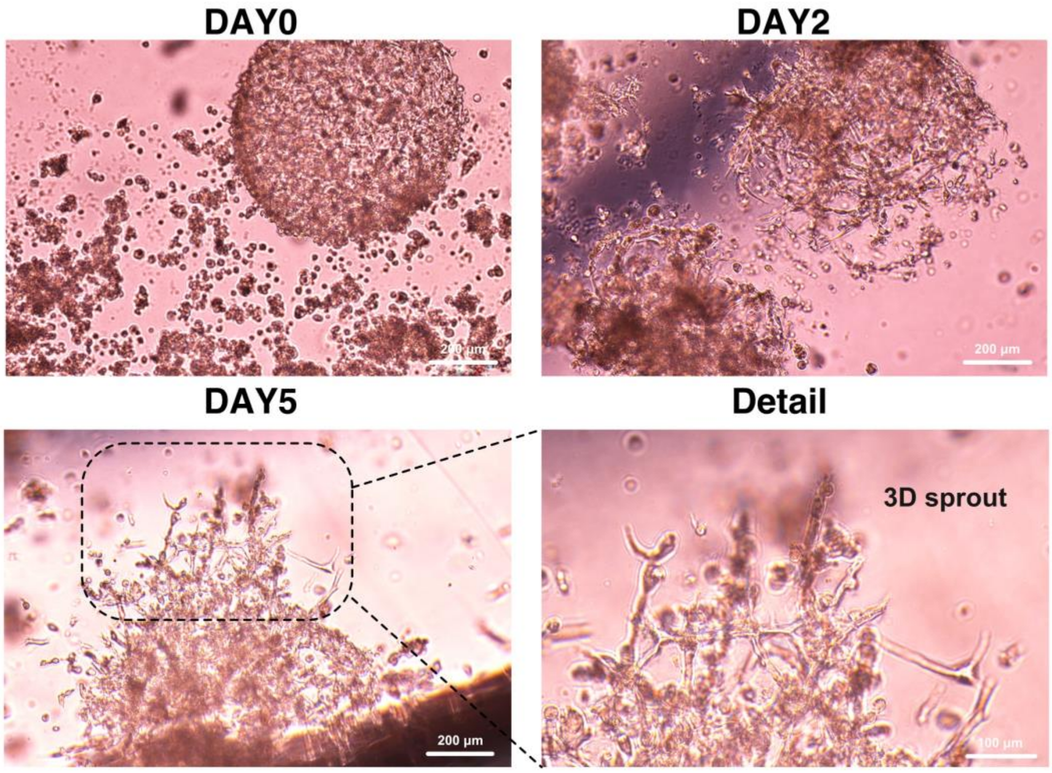

Supplementary Fig. 12 | Co-culturing and vascularization of HUVECs/MDA-MB-231s-laden A-C structure. Each experiment was repeated independently with similar results for 3 times.

Supplementary Note 9 | Diameter of extruded A-C bioink filament

A30/5-C30/20 and A30/5-C300/20 were extruded from 20G cone-shape nozzle at 150 μL/min, respectively. The moving speeds of nozzle were set as 80 mm/min, 120 mm/min and 180 mm/min. The bioink temperatures were set as 4 ℃, 24 ℃, 37 ℃. The diameters of extruded filaments were measured with optical microscopy (Supplementary Fig. 13). At 80 mm/min, A-C bioink tended to accumulate around the nozzle because of the slow movement of printing nozzle. At 180 mm/min, A-C bioink tended to be stretched or even snapped accumulate around the nozzle because of the fast movement of printing nozzle. At 120 mm/min, the filament diameters tended to be uniform under all temperatures. Therefore, 120 mm/min was regarded as promising nozzle moving speed for 20G nozzle and 150 μL/min bioink flowrate.

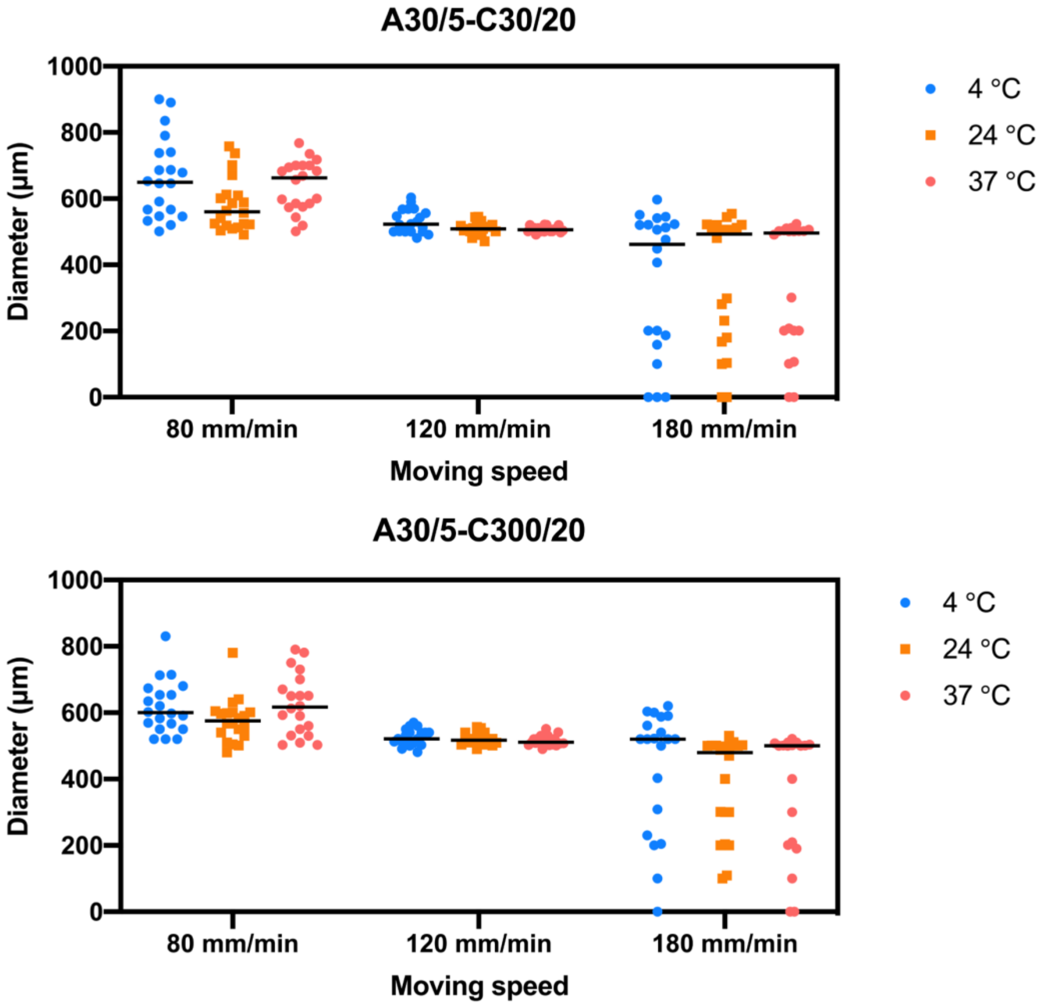

Supplementary Fig. 13 | Diameter of extruded A-C bioink filament
