# Supplementary material for: In situ 3D bioprinting with bioconcrete bioink
Source: Nat Commun. 2022 Jun 23;13:3597. doi: 10.1038/s41467-022-30997-y (PMC9225998; doi:10.1038/s41467-022-30997-y)
Supplement: Supplementary file 3 — Description of Additional Supplementary Files [file 41467_2022_30997_MOESM3_ESM.docx]

**Description of Additional Supplementary Files**

**Supplementary Video. 1 | Comparison of fluidity of A-C bioink and C bioink in 37 ℃.** Unlike C bioink with high fluidity, A-C bioink can maintain both fluidity and viscosity even in such a high temperature, which makes it owns the printability in in-situ bioprinting.

**Supplementary Video. 2 | State of extruding fiber of A-C bioink.** A-C bioink can form uniform in-situ bioprinting filament in such a high temperature, which makes it owns the printability in in-situ bioprinting.

**Supplementary Video. 3 |** Extruding bioprinting with A-C bioink in simulated in-situ condition. A simulated in-situ bioprinting scene was established to evaluate the printability of A-C bioink. A-C bioink was successfully printed with a commercial 3D bioprinter to establish a 3D cube with 12 layers and 7.5 mm height in the roughly controlled environment temperature (30 ℃) and on the “wound” receiving basement.

**Supplementary Video. 4 |** Binding force on the interface between tissue and A-C composite structure. To distinctly observe the strong binding force, A30/5-C30/20 bioink was poured on fresh pig tendon, while A30/5-C300/20 bioink was poured on fresh pig rib. All-direction forces were added to A-C structures. A-C structures strongly attached to the tissue surface.

**Supplementary Video. 5 |** In-situ bioprinting of cranial defect of rat patients with A-C bioink. The robotic arm system was selected as the in-situ bioprinting tool. Rat “patient” was placed on the operating table. The 3D model of the defect was rebuilt with computer-aided design software, and the printing routine program was generated with slicing software and loaded into the controlling system of the robotic arm. A30/5-C300/20 bioink encapsulated BMSCs were in-situ deposited into the cranial defect of four “patients” and photocrosslinked with 405-nm blue light.
