# Supplementary material for: In situ 3D bioprinting with bioconcrete bioink
Source: Nat Commun. 2022 Jun 23;13:3597. doi: 10.1038/s41467-022-30997-y (PMC9225998; doi:10.1038/s41467-022-30997-y)
Supplement: Supplementary file 9 — Reporting Summary [file 41467_2022_30997_MOESM9_ESM.pdf]

nature portfolio

Corresponding author(s): Yong He

Last updated by author(s): May 19, 2022

Reporting Summary

Nature Portfolio wishes to improve the reproducibility of the work that we publish. This form provides structure for consistency and transparency in reporting. For further information on Nature Portfolio policies, see our Editorial Policies and the Editorial Policy Checklist.

Statistics

For all statistical analyses, confirm that the following items are present in the figure legend, table legend, main text, or Methods section.

n/a	Confirmed	
☐	☒	The exact sample size (*n*) for each experimental group/condition, given as a discrete number and unit of measurement
☐	☒	A statement on whether measurements were taken from distinct samples or whether the same sample was measured repeatedly
☒	☐	The statistical test(s) used AND whether they are one- or two-sided<br>*Only common tests should be described solely by name; describe more complex techniques in the Methods section.*
☒	☐	A description of all covariates tested
☒	☐	A description of any assumptions or corrections, such as tests of normality and adjustment for multiple comparisons
☐	☒	A full description of the statistical parameters including central tendency (e.g. means) or other basic estimates (e.g. regression coefficient) AND variation (e.g. standard deviation) or associated estimates of uncertainty (e.g. confidence intervals)
☒	☐	For null hypothesis testing, the test statistic (e.g. *F*, *t*, *r*) with confidence intervals, effect sizes, degrees of freedom and *P* value noted<br>*Give P values as exact values whenever suitable.*
☒	☐	For Bayesian analysis, information on the choice of priors and Markov chain Monte Carlo settings
☒	☐	For hierarchical and complex designs, identification of the appropriate level for tests and full reporting of outcomes
☒	☐	Estimates of effect sizes (e.g. Cohen's *d*, Pearson's *r*), indicating how they were calculated

Our web collection on statistics for biologists contains articles on many of the points above.

Software and code

Policy information about availability of computer code

Data collection	Zen (Carl Zeiss, version 8,0,0,273) , COMSOL Multiphysics 5.4 (COMSOL AB.), Repetier (Window 2.2.4, Hot-World GmbH & Co KG)
Data analysis	MATLAB 2021 (MathWorks), GraphPad Prism 8 (GraphPad Software), ImageJ (Java 1.8.0_172), FlowJo (version 10.4 for mac, Becton Dickinson&Company)

For manuscripts utilizing custom algorithms or software that are central to the research but not yet described in published literature, software must be made available to editors and reviewers. We strongly encourage code deposition in a community repository (e.g. GitHub). See the Nature Portfolio guidelines for submitting code & software for further information.

Data

Policy information about availability of data

All manuscripts must include a data availability statement. This statement should provide the following information, where applicable:

- Accession codes, unique identifiers, or web links for publicly available datasets
- A description of any restrictions on data availability
- For clinical datasets or third party data, please ensure that the statement adheres to our policy

The authors declare that all data supporting the findings of this study are available within the paper and its Supplementary Information or from the corresponding authors upon request.

Field-specific reporting

Please select the one below that is the best fit for your research. If you are not sure, read the appropriate sections before making your selection.

☒ Life sciences ☐ Behavioural & social sciences ☐ Ecological, evolutionary & environmental sciences

For a reference copy of the document with all sections, see nature.com/documents/nr-reporting-summary-flat.pdf

Life sciences study design

All studies must disclose on these points even when the disclosure is negative.

Sample size	For cell experiments, more than 30 cell-laden microgels for different testing items at different time points were used, respectively. For the bone regeneration in cranial defects experiment, STATA 12.0 software (StataCorp, TX, USA) was used to estimate sample sizes. Sample sizes were calculated based on alpha=0.05 and power=0.80. 5 rats were used for 3 groups and 2 time points, respectively. For the in-situ bioprinting experiment, 4 rats with different defect morphologies were used. All of the sample size designs were sufficient to demonstrate the capabilities of the bioink.
Data exclusions	No data were excluded from analysis.
Replication	All of the in-vitro experients were performed for three times.
Randomization	All rats used in the study were assigned randomly to experimental groups using randomization lists generated from PASS 11.0 software (NCSS, UT, USA).
Blinding	Some measurements and calculations were conducted by an investigator who was blinded to grouping and treatment information, including micro-computed tomography analysis.

Reporting for specific materials, systems and methods

We require information from authors about some types of materials, experimental systems and methods used in many studies. Here, indicate whether each material, system or method listed is relevant to your study. If you are not sure if a list item applies to your research, read the appropriate section before selecting a response.

Materials & experimental systems

n/a	Involved in the study	
☐	☒	Antibodies
☐	☒	Eukaryotic cell lines
☒	☐	Palaeontology and archaeology
☐	☒	Animals and other organisms
☒	☐	Human research participants
☒	☐	Clinical data
☒	☐	Dual use research of concern

Methods

n/a	Involved in the study	
☒	☐	ChIP-seq
☐	☒	Flow cytometry
☒	☐	MRI-based neuroimaging

Antibodies

Antibodies used	TRITC Phalloidin (40734ES75, Yeasen Biotechnology)
Validation	TRITC Phalloidin (40734ES75, Yeasen Biotechnology) was validated in cell (any animals and plants) staining and immunofluorescence.

Eukaryotic cell lines

Policy information about cell lines

Cell line source(s)	BMSCs (primary cells from rats) were provided by Stomatology Hospital, School of Stomatology, Zhejiang University School of Medicine, Zhejiang Provincial Clinical Research Center for Oral Diseases, Key Laboratory of Oral Biomedical Research of Zhejiang Province, Cancer Center of Zhejiang University, Hangzhou 310006. MDA-MB-231s, HUVECs, and MC3T3-E1s were purchased from Shanghai Zhongqiaoxinzhou Biotech.
Authentication	None of the cell lines are authenticated.
Mycoplasma contamination	All cell lines were tested negative for mycoplasma contamination.
Commonly misidentified lines (See ICLAC register)	No commonly misidentified lines were used.

Animals and other organisms

Policy information about studies involving animals; ARRIVE guidelines recommended for reporting animal research

Laboratory animals	12-week old male SD rate (250-300 g). The temperature of the rearing room was kept at 20~25℃, and the relative humidity was 50% ~65%, with 12h light and 12h dark day/night cycle provided.
Wild animals	The study did not involve wild animals.
Field-collected samples	The study did not involve field-collected samples.
Ethics oversight	The experiments were approved by the Ethics Committee for Animal Research at Zhejiang University (ethics approval number: ZJU20210172) and performed in accordance with the Institutional Animal Care and Use Committee of Zhejiang University.

Note that full information on the approval of the study protocol must also be provided in the manuscript.

Flow Cytometry

Plots

Confirm that:

- [x] The axis labels state the marker and fluorochrome used (e.g. CD4-FITC).
- [x] The axis scales are clearly visible. Include numbers along axes only for bottom left plot of group (a 'group' is an analysis of identical markers).
- [x] All plots are contour plots with outliers or pseudocolor plots.
- [x] A numerical value for number of cells or percentage (with statistics) is provided.

Methodology

Sample preparation	The A30/5 with BMSCs were electrosprayed as above, half of which were extruded from 20G cone-shape nozzle at 150 µL/min. After 4-hour culturing, the extruded and non-extruded A30/5 with BMSCs was degraded with 20 U/mL collagenase II PBS solution for 30 min to remove GelMA hydrogel. The harvested cells were stained with Annexin V-FITC/PI kits and tested by flow cytometry, respectively. The data were analyzed with FlowJo software.
Instrument	FACS Caliber (BD)
Software	FlowJo (version 10.4 for mac, Becton Dickinson&Company)
Cell population abundance	More than 1e5 cells in each sample.
Gating strategy	-Excluding cell aggregate and fragment according to FSC&SSC;<br>-Negative&compensation (single staining respectively) control were set.<br>-Setting crossed door based on no extrusion group.

- [x] Tick this box to confirm that a figure exemplifying the gating strategy is provided in the Supplementary Information.
